# No differences in brain microstructure between young KIBRA-C carriers and non-carriers

**DOI:** 10.18632/oncotarget.23348

**Published:** 2017-12-16

**Authors:** Li Hu, Qunxing Xu, Jizhen Li, Feifei Wang, Xinghua Xu, Zhiyuan Sun, Xiangxing Ma, Yong Liu, Qing Wang, Dawei Wang

**Affiliations:** ^1^ Department of Radiology, Qilu Hospital of Shandong University, Jinan 250012, China; ^2^ Medical Examination Center, Qilu Hospital of Shandong University, Jinan 250012, China; ^3^ Mental Health Center of Shandong Province, Jinan 250012, China; ^4^ Brainnetome Center, National Laboratory of Pattern Recognition, Institute of Automation, Chinese Academy of Sciences, Beijing 100190, China

**Keywords:** DKI, KIBRA, brain microstructure, young

## Abstract

KIBRA rs17070145 polymorphism is associated with variations in memory function and the microstructure of related brain areas. Diffusion kurtosis imaging (DKI) as an extension of diffusion tensor imaging that can provide more information about changes in microstructure, based on the idea that water diffusion in biological tissues is heterogeneous due to structural hindrance and restriction. We used DKI to explore the relationship between KIBRA gene polymorphism and brain microstructure in young adults. We recruited 100 healthy young volunteers, including 53 TT carriers and 47 C allele carriers. No differences were detected between the TT homozygotes and C-allele carriers for any diffusion and kurtosis parameter. These results indicate KIBRA rs17070145 polymorphism likely has little or no effect on brain microstructure in young adults.

## INTRODUCTION

KIBRA gene, also known as WWC1 gene located on chromosome 5q35.1, was first described in 2003 [1]. Gene expression and immunohistological studies showed that KIBRA is expressed in memory-related regions of the brain, such as the hippocampus and cortex, as well as in the cerebellum and the hypothalamus [2, 3]. At those locations, KIBRA is involved in cellular functions such as synaptogenesis, vesicle transport, transcriptional regulation, cell polarity and migration, and it plays an important role in synaptic plasticity and human memory performance and cognition [1–5].

Genome-wide analyses have shown that KIBRA rs17070145 single nucleotide polymorphism (T-C substitution) is significantly associated with memory performance [6]. Follow-up studies have reported the KIBRA rs17070145 is associated with episodic memory function and cognitive flexibility [7–10]. Overall, KIBRA CC homozygotes showed poorer episodic memory performance than T-carriers among healthy subjects. However, two studies failed to confirm an association between the KIBRA polymorphism and episodic memory [11, 12]. Genetic variation in KIBRA has also been linked to the risk for late-onset Alzheimer’s disease (AD), which is associated with severe episodic memory impairment [13–16]. Notably, KIBRA T allele carriers were reportedly at significantly less risk of developing AD [15]. On the other hand, the T allele increased the risk of very-late-onset (after the age of 86 years) AD [13]. A recent meta-analysis indicated that the KIBRA T allele had a protective effect, though its impact on AD risk is modest [14].

The effects of KIBRA polymorphism on brain function have been investigated in several neuroimaging studies. In a task-based fMRI study, KIBRA CC carriers presented higher brain activation than T allele carriers in memory-related brain regions, especially the hippocampus and parahippocampal gyrus [6]. Another fMRI study in healthy older subjects showed that in KIBRA CC homozygotes, activation in the hippocampal region was lower than in T carriers [17]. In a positron emission tomography (PET) study of cognitively normal late-middle-aged persons, KIBRA rs17070145 CC carriers showed lower glucose metabolism than T-carriers in AD-related brain regions [15]. Through region of interest (ROI)-based analyses, Witte *et al.* found that KIBRA T-allele carriers had significantly higher hippocampal volume but lower functional connectivity of the left hippocampus [18]. We recently observed in a healthy young cohort that KIBRA C-allele carriers show higher synchronization in the regions of the default mode network (DMN) and executive control network (ECN) than TT carriers [19]. To our knowledge, there has been no study to determine how the KIBRA rs17070145 polymorphism affects the brain microstructure using diffusion kurtosis imaging (DKI) in healthy young adults.

DKI is an extension of diffusion tensor imaging (DTI) that is based on the theory that water diffusion in biological tissues is distribution in non-Gaussian fashion due to structural hindrance and restriction. It is sensitive to tissue microstructure and provides more information about the changes of microstructure. Based on DKI data, both diffusion and kurtosis parameters, including mean kurtosis (MK), axial kurtosis (AK) and radial kurtosis (RK), can be obtained [20–22]. The kurtosis parameters are especially suitable for evaluating microstructural integrity in regions with complex fiber arrangement and isotropic tissues, such as gray matter [23–25]. DKI exhibits improved sensitivity and specificity for detecting developmental and pathological changes in neural tissues and is widely used to assess demyelinating diseases, epilepsy, AD, Parkinson’s disease and Huntington’s chorea, among others [26, 27].

In the present study, we compared all the diffusion and kurtosis parameters between KIBRA C and TT allele carriers in an effort to explore the relationship between KIBRA gene polymorphism and brain microstructure among healthy young adults.

## RESULTS

### Subjects

100 healthy young Chinese Han subjects with high-quality imaging data and KIBRA genotypic information were finally included in this study. These subjects were divided into two groups including 53 TT homozygotes and 47 C-allele carriers (37 CT and 10 CC carriers) according to the KIBRA genotypes which was consistent with the previous studies [19, 28]. There were no significant differences (*p* > 0.05) in age, gender, years of education, between the two groups (Table 1).

**Table 1:** Demographic data of subjects

	CC+CT	TT	*P* value
Number of subjects	47	53	
age (years)	25.34 (1.54)	25.95 (1.75)	0.089
Gender (M/F)	17/30	23/30	0.462
Years of education	17.95 (0.27)	18.08 (0.23)	0.644

Data are presented as the mean ± standard deviation (SD). Abbreviation: M, male; F, female.

### Group analysis of DKI parameters

No significant differences was found between KIBRA TT homozygotes and C-allele carriers in all diffusion and kurtosis parameters with *p* value < 0.05 (FDR cor). However, we investigated several differences between the two groups using a relatively loose threshold (*p* < 0.001 uncorrected with a minimum cluster size 10).

### Group differences in whiter matter (WM)

**Figure 1:**
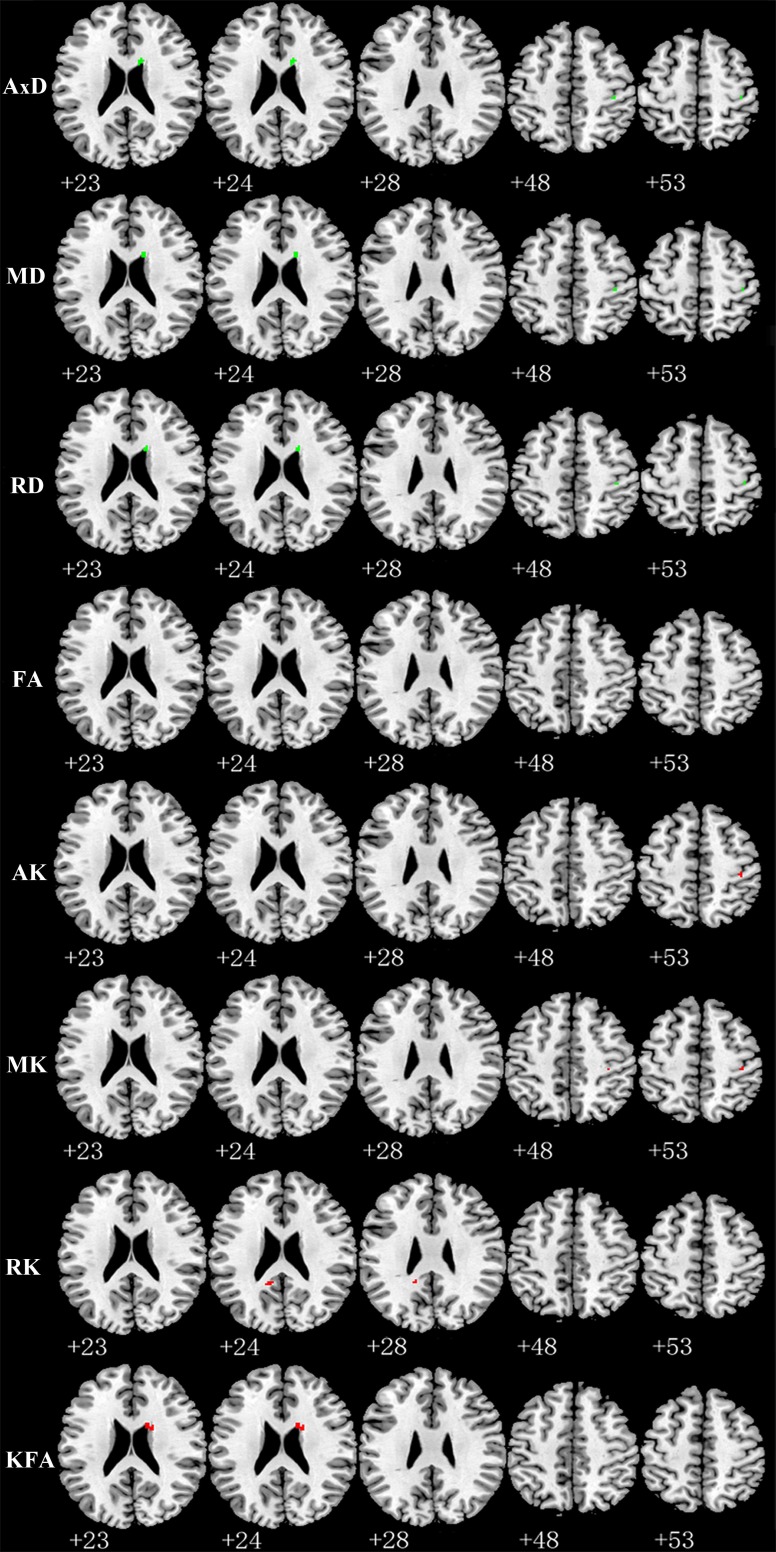
Representative axial images show differences in WM (*p* < 0.001, with a minimum cluster size 10) between TT carriers and C carriers for all indices: AxD, MD, RD, FA, AK, MK, RK and KFA Green and red represents the clusters with increased and decreased indices in KIBRA C-allele carriers compared with TT carriers, respectively. FA showed no difference between the two groups. Abbreviation: AxD: axial diffusivity; MD: mean diffusivity; RD: radial diffusivity; FA: fractional anisotropy; AK: axial kurtosis; MK: mean kurtosis; RK: radial kurtosis; KFA: kurtosis fractional anisotropy.

Compared with TT homozygotes, KIBRA C-allele carriers showed increased AxD (axial diffusivity), MD (mean diffusivity), RD (radial diffusivity), decreased AK and MK in right postcentral gyrus WM. And we found increased AD, MD, RD and decreased KFA (kurtosis fractional anisotropy) in right periventricular WM in KIBRA C-allele carriers. (Figure 1). The results are listed in Table 2.

**Table 2:** WM regions showed differences in indices: AxD, MD, RD, AK, KFA, MK and RK between KIBRA TT carriers and C carriers

Index		Peak MNI Coordinate region	Peak MNI coordinates			Number of cluster voxels	Peak *t* value
			x	y	z		
AxD	1	Right Postcentral Gyrus wm	38	−27	50	11	3.95
	2	Right Lateral Ventricle wm	12	12	20	27	4.05
MD	1	Right Postcentral Gyrus wm	38	−27	50	14	3.97
	2	Right Lateral Ventricle wm	16	12	20	22	3.91
RD	1	Right Postcentral Gyrus wm	38	−27	52	12	3.88
	2	Right Lateral Ventricle wm	16	12	20	18	3.98
AK	1	Right Postcentral Gyrus wm	40	−27	56	11	3.90
	2	Left Precentral Gyrus wm	−40	−11	34	12	5.21
KFA	1	Right Lateral Ventricle wm	20	4	22	28	4.02
MK	1	Right Postcentral Gyrus wm	40	−25	56	19	3.96
	2	Right Medial Frontal Gyrus wm	14	40	32	11	3.91
RK	1	Left Cingulate Gyrus wm	−14	−45	26	16	3.75

Abbreviation: AxD: axial diffusivity; MD: mean diffusivity; RD: radial diffusivity; FA: fractional anisotropy; AK: axial kurtosis; MK: mean kurtosis; RK: radial kurtosis; KFA: kurtosis fractional anisotropy.

### Group differences in gray matter (GM)

**Figure 2:**
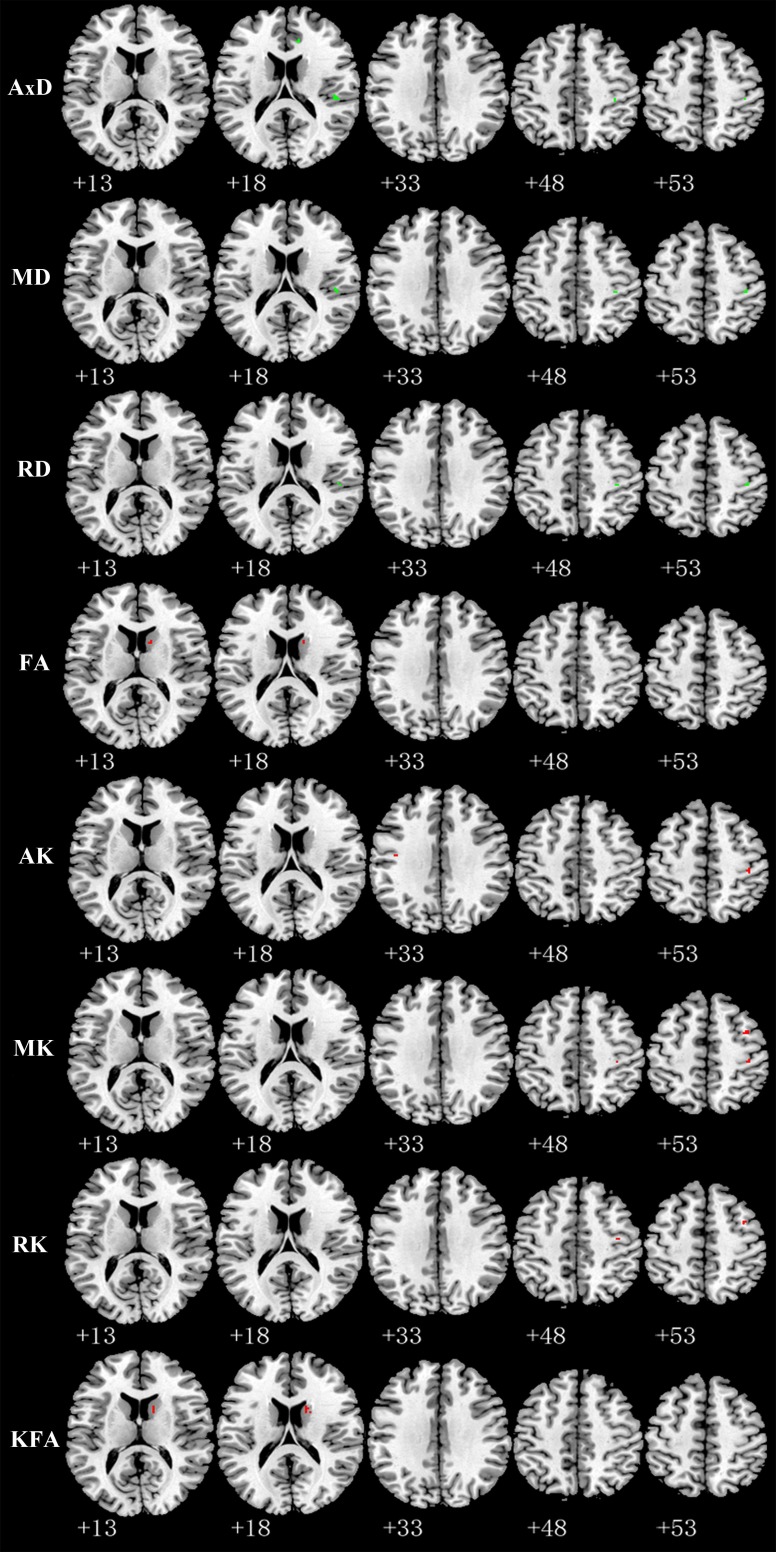
Representative axial images show GM regions differences (*p* < 0.001,with a minimum cluster size 10) between TT carriers and C carriers for all indices: AxD, MD, RD, FA, AK, MK, RK, and KFA Green and red represents the clusters with increased and decreased indices in KIBRA C-allele carriers compared with TT carriers, respectively. Abbreviation: AxD: axial diffusivity; MD: mean diffusivity; RD: radial diffusivity; FA: fractional anisotropy; AK: axial kurtosis; MK: mean kurtosis; RK: radial kurtosis; KFA: kurtosis fractional anisotropy.

**Figure 3:**
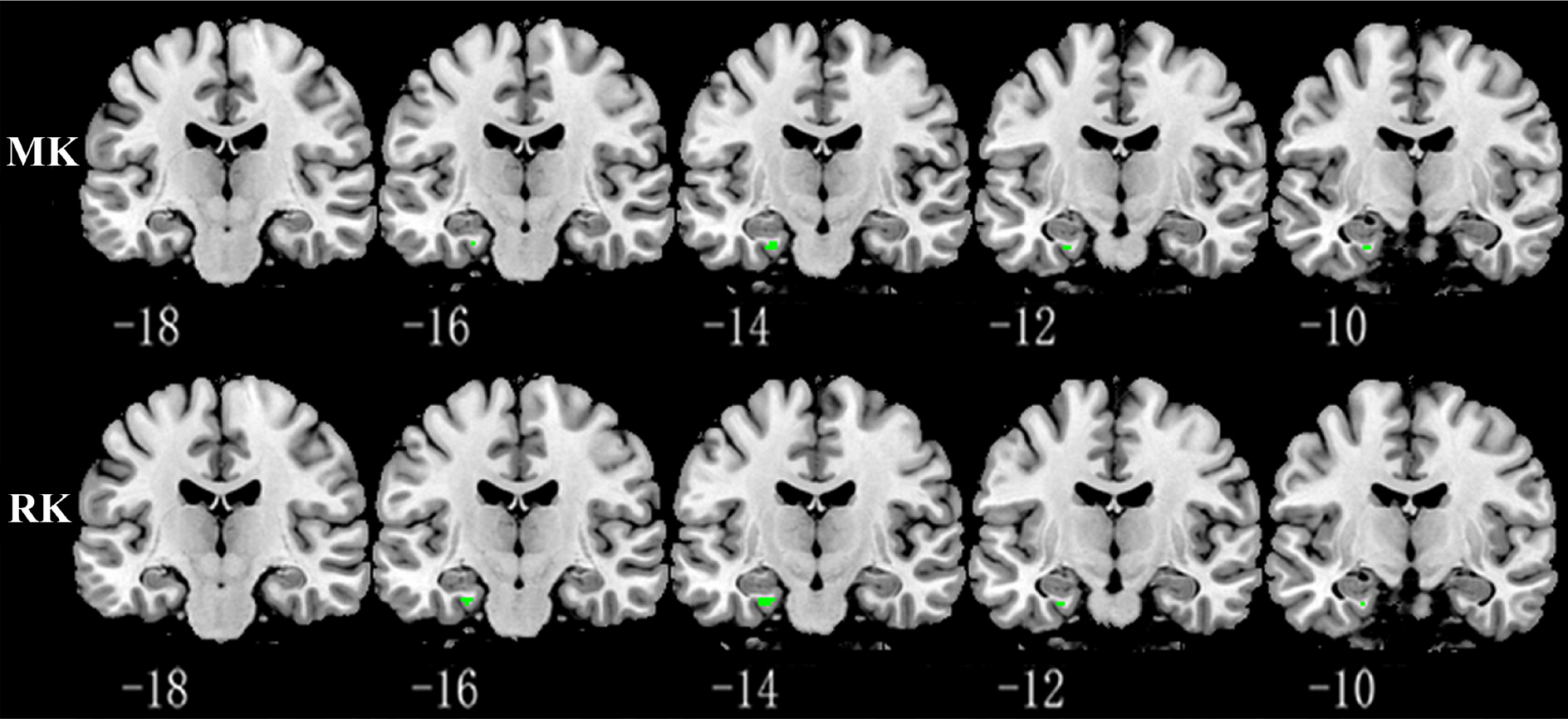
Representative coronal images show increased MK and RK in parahippocampus (*p* < 0.001, with a minimum cluster size 10) in KIBRA C-allele carriers compared with TT carriers Abbreviation: MK: mean kurtosis; RK: radial kurtosis.

Relative to TT homozygotes, KIBRA C-allele carriers showed increased AxD, MD, RD in right postcentral gyrus and right insula, decreased AK, MK in right postcentral gyrus, as well as decreased FA (fractional anisotropy), KFA in right caudate head (Figure 2). The results are listed in Table 3. Moreover, KIBRA C-allele carriers showed increased MK and RK in the left parahippocampus, compared to TT homozygotes (Figure 3). The results are listed in Table 4.

**Table 3:** GM regions showed differences in indices: AxD, MD, RD, FA, AK, KFA, MK and RK between KIBRA TT carriers and C carriers

Index		Peak MNI coordinate region	Peak MNI coordinates			Number of cluster voxels	Peak *t* value
			x	y	z		
AxD	1	Right Postcentral Gyrus	38	−27	50	12	3.94
	2	Right Insula	48	−28	20	17	4.18
	3	Right Anterior Cingulate	10	34	20	16	4.20
MD	1	Right Postcentral Gyrus	38	-27	50	14	3.94
	2	Right Insula	48	-30	20	16	4.11
RD	1	Right Postcentral Gyrus	38	−27	52	12	3.88
	2	Right Insula	48	−28	20	14	4.04
	3	Lingual Gyrus	6	−82	−10	10	3.91
FA	1	Right Caudate Head	14	10	16	12	4.49
AK	1	Right Postcentral Gyrus	40	-27	56	11	3.90
	2	Left Precentral Gyrus	-40	-11	34	10	5.21
KFA	1	Right Caudate Head	20	6	20	39	4.02
MK	1	Right Postcentral Gyrus	40	-25	56	19	3.96
RK	2	Right Middle Frontal Gyrus	36	2	54	13	3.80
	1	Right Precentral Gyrus	38	-13	48	10	3.48
	2	Right Middle Frontal Gyrus	36	4	54	12	3.87
	3	Left Precuneus	-20	-65	42	10	3.77

Abbreviation: AxD: axial diffusivity; MD: mean diffusivity; RD: radial diffusivity; FA: fractional anisotropy; AK: axial kurtosis; MK: mean kurtosis; RK: radial kurtosis; KFA: kurtosis fractional anisotropy.

**Table 4:** GM regions showed increased MK and RK in parahippocampus in KIBRA C-allele carriers compared with TT carriers

Index		Peak MNI coordinate region	Peak MNI coordinates			Number of cluster voxels	Peak t value
			x	y	z		
MK RK	1 2	Left Parahippocampa Gyrus Left Parahippocampa Gyrus	−24 −24	−14 −14	−28 −28	10 14	−3.83 −4.00

Abbreviation: MK: mean kurtosis; RK: radial kurtosis.

## DISCUSSION

To our knowledge, this is the first research using DKI to investigate the effect of KIBRA polymorphism on brain microstructure in healthy young subjects. The study showed no significant difference between KIBRA TT homozygotes and C-allele carriers for any diffusion and kurtosis parameter. However, we investigated several differences between the two groups using a relatively loose threshold. These findings suggest that KIBRA polymorphism likely has little or no influence on brain microstructure in healthy young adults.

Gene expression studies demonstrated that KIBRA is highly expressed in the frontal, parietal and temporal lobes, which are thought to be memory-related brain regions and are more easily impaired than other brain parts [6, 29]. Therefore, the effects of the KIBRA polymorphism on brain functions have been investigated in several neuroimaging studies. A task-based fMRI study recruiting 15 T carriers and 15 CC carriers demonstrated that KIBRA CC carriers show higher brain activation than T allele carriers in memory-related brain regions (especially the hippocampus and parahippocampal gyrus) [6]. The CC carriers needed more brain activation to perform as well as the T carriers. Another fMRI study including 83 samples from 55- to 60-year-old individuals without dementia showed that KIBRA CC homozygotes had lower hippocampal activation than T carriers [17]. In a PET-CT study of cognitively normal late-middle-aged persons incorporating 67 CC carriers and 69 T carriers, KIBRA rs17070145 CC carriers showed lower glucose metabolism than T-carriers in AD-related brain regions (posterior cingulate and precuneus regions) [15]. The findings in the healthy old were opposite to those in the young. One possible explanation for this is that KIBRA-C carriers require more brain activity in memory-related brain regions to maintain normal cognition in the young, but this compensatory response is lost by the time individuals reach later stages of life. As a result, KIBRA-C carriers show poorer cognitive performance.

In our study, there was no significant difference between KIBRA TT homozygotes and C-allele carriers in any diffusion or kurtosis parameter. Histologic examinations in brains from AD model mice and human studies suggest increased diffusion parameters with decreased FA reflect demyelination and loss of axons in whiter matter. The decreased kurtosis parameters reflected the loss of neuron cell bodies, synapses and dendrites in gray matter [30–32]. In the young, the KIBRA polymorphism has no or little effect on brain microstructure. DKI parameters may not be sensitive enough to detect the brain microstructural differences between KIBRA TT homozygotes and C-allele carriers, although brain function has changed. The APOE 4 allele is associated with a high risk for AD [33], and several studies have investigated the effects of APOE polymorphism on brain microstructure. One study including 575 healthy adolescents found no difference in white matter microstructural organization between carriers and non-carriers of the APOE 4 allele, based on diffusion tensor imaging parameters [34]. Another study incorporating 1412 healthy adolescents reported no differences in hippocampal volume between APOE 4 carriers and non-carriers were found [35]. However, a study including 44 healthy subjects between 20 and 38 years of age found that hippocampal volume was reduced in healthy young APOE 4 carriers relative to non-carriers [36]. Moreover, a majority of studies in older individuals and AD patients demonstrated that APOE 4 carriers show greater rates of temporal lobe atrophy [37, 38]. Lindenberger and colleagues hypothesized that genetic effects in younger adults may be small or undetectable, but these effects become more pronounced as people age [39]. Similarly, the effects of KIBRA rs17070145 polymorphism may not be apparent until later adulthood.

Using a relatively loose threshold, we found increased AxD, MD, RD, decreased AK and MK in right postcentral gyrus, which is a key region of the sensorimotor network. In addition, there is increasing evidence for sensorimotor dysfunction early in the disease [40]. For example, in healthy young subjects, APOE ε4 carriers exhibited increased functional connectivity in the sensorimotor network [41]. Evidence from behavioral studies in elderly individuals indicates that APOE ε4 carriers have greater vulnerability for impaired motor function [42]. fMRI studies have revealed that sensorimotor network connectivity is decreased or rewired in AD [43]. A resting-state network study revealed that the sensorimotor network exhibits decreased connectivity in healthy elders with APOE ε4 [44]. The increased AxD, MD, RD, decreased AK and MK in the right postcentral gyrus might be an indicator for the impairment of the regional microstructure.

Another interesting finding was that KIBRA C allele carriers showed greater MK and RK in the left parahippocampus than TT homozygotes. In contrast to conventional DTI parameters, MK and RK are regarded as indices of the complexity of tissue microstructure, reflecting the density, orientation, and degree of organization of cell membranes, axon sheaths, and myelin layers [20, 45–47]. Using diffusion kurtosis imaging with a cohort the included individuals with AD or mild cognitive impairment (MCI) and normal controls, Gong *et al.* recently reported significantly decreased hippocampal MK in MCI and AD, which reflects the loss of microstructural compartments such as neuronal cell bodies, axons, synapses, and dendrites. In addition, using resting fMRI, Filippini *et al.* [48] detected greater default mode network synchronization involving medial temporal and medial-prefrontal cortical areas in APOE ε4-carriers than noncarriers. Our results are, to some extent, consistent with these studies. Increased MK and RK in the left parahippocampus in healthy young KIBRA C allele carriers likely reflects a compensatory mechanism to maintain relatively normal memory function.

There are several limitations to the present study. First, the small number of CC-individuals (10/100) made our grouping method different from most studies, though our previous studies suggest combining the CC and CT individuals is reasonable, at least in the Chinese population. A much larger sample should be collected in the future, especially KIBRA CC homozygotes. Second, other gene polymorphisms associated with memory or cognitive function (e.g. BDNF and CLSTN2) were not controlled in this study, which may bias our results. Finally, we mainly focused on microstructural changes in healthy young adults. This same approach could be used to determine whether an effect of KIBRA polymorphism on brain microstructure is more apparent in cognitively intact older adults and in AD patients.

In summary, no significant differences were investigated between KIBRA rs17070145 TT homozygotes and C-allele carriers in all diffusion and kurtosis parameters in healthy young adults. And these results suggest that KIBRA rs17070145 polymorphism might have no or slight effect on brain microstructure of young. However, the effect of KIBRA rs17070145 polymorphism on brain microstructure may occur or become more obvious later in life.

## MATERIALS AND METHODS

### Subjects

A total of 125 healthy young Chinese subjects (mean age: 25 ± 1.54 years; ranging 23–28 years; 68 females, 57 males;) were recruited in this study after giving written informed consent, in accordance with Medical Ethics Committee of Qilu Hospital of Shandong University. Careful screening was performed to ensure that all participants were free of any lifetime history of psychiatric or neurological illness, psychiatric treatment, or drug or alcohol abuse, and MR contraindications. To avoid population stratification artifacts, only Chinese Han subjects were included. Finally, we recruited 100 healthy young with right-handed subjects from 125 subjects. Twenty-five subjects were excluded from further study owing to lack of genetic data or poor imaging during the MRI examinations.

### Genotyping

Fasting venous blood was collected from the peripheral vein. DNA was extracted using a Puregene kit (Gentra Systems, Inc., Minneapolis, MN, USA). Quality control was performed at the DNA-sample level, assay level, and the level of multiplex assay pool. The call rates for the SNPs included in this study were between 99.4 and 99.7%. KIBRA rs17070145 was genotyped using the Sequenom Mass Array system (Sequenom Inc., San Diego, CA, USA) with technical support from CapitalBio Technology (Beijing, China). Mass determination was carried out with the MALDI-TOF mass spectrometer and Mass ARRAY Type 4.0 software was used for data acquisition.

Among individuals included in the study, frequencies of genotypes in the KIBRA rs17070145 polymorphism were T/T in 53 subjects (53%), C/T in 37 subjects (37%), and C/C in 10 subjects (10%). Allelic frequencies were in line with previous reports and Hardy Weinberg Equilibrium was *p* = 0.62 for rs17070145.

### Image acquisition

Images were acquired in a Siemens verio 3.0 Tesla MR scanner (Erlangen, Germany) with a 8-channel head coil with soft foam padding. DKI images were acquired with 3 *b*-values (*b* = 0, 1000 and 2000 s/mm^2^) along 20 diffusion gradient directions using a single-shot, spin-echo EPI sequence. Other imaging parameters were: TR = 9600 ms, TE = 96 ms; FOV = 256 × 256 mm^2^; matrix = 128 × 128; voxel = 2× 2 × 3 mm^3^; 45 axial slices；slice thickness 3.0 mm. Sagittal 3D T1-weighted images were acquired by magnetization prepared rapid acquisition gradient echo (MPRAGE) sequence (TR/TE = 2000/2.3 ms; inversion time = 900 ms; FA = 9°; matrix = 256 × 256; slice thickness = 1 mm, no gap; 192 slices).

### Data analysis

Eddy current-induced distortion and motion artifacts in the DKI dataset were corrected using affine alignment of each diffusion weighted image to the *b* = 0 image using FMRIB’s diffusion toolbox (FSL 4.0, http://www.fmrib.ox.ac.uk/fsl) [49]. After skull-stripping, Diffusional Kurtosis Estimator (http://www.nitrc.org/projects/dke) was implemented to calculate the diffusion and kurtosis tensors including mean kurtosis (MK), axial kurtosis (AK), radial kurtosis (RK), kurtosis fractional anisotropy (KFA) as well as fractional anisotropy (FA), mean diffusivity (MD), axial diffusivity (AxD), and radial diffusivity (RD). Voxel-based analysis was as follows: Firstly, all the b0 (*b* = 0) images were normalized to the EPI template using coregistration tool in the SPM8 (http://www.fil.ion.ucl.ac.uk/spm/), with a reslicing resolution of 2 × 2 × 2 mm^3^. Afterwards, these normalized b0 images were averaged and smoothed with a full width at half maximum (FWHM) kernel of 6 × 6 × 6 mm^3^ to generate a new subject-specific b0 template. Secondly, all the b0 images were again normalized to the new b0 template to get the transformation information, which was then applied to all of the corresponding parametric maps. Then all the parametric maps were smoothed using a FWHM kernel of 6 × 6 × 6 mm^3^ to reduce possible error in local anatomy. For each subject, the sagittal T1-weighted image was segmented into GM, WM and cerebrospinal fluid in voxel-based morphometry (VBM8, http://dbm.neuro.uni-jena.de/vbm8/), which were then normalized to the standard Montreal Neurological Institute (MNI) space using SPM8 with a reslicing resolution of 2 × 2 × 2 mm^3^. The normalized GM and WM images for each subject were averaged and smoothed with a 6 mm FWHM Gaussian kernel, the mean of which, including all voxels with a GM probability greater than 0.3, WM greater than 0.4 were converted into masks.

### Statistical analysis

Group comparisons of these data were performed using a χ^2^ test for categorical variables and *t*-tests for continuous variables. Significance level was set at *p* < 0.05 with two-tailed tests. Two-sample tests were applied to detect WM and GM differences in all diffusion and kurtosis parameters between TT homozygotes and C-allele carriers with *p* value < 0.05, False Discovery Rate (FDR) corrected.
